# THE ROLE OF SOCIAL SUPPORT AND SELF-EFFICACY ON HOPEFULNESS IN LOW-INCOME OLDER ADULTS AGED 50 AND OVER

**DOI:** 10.1093/geroni/igad104.1997

**Published:** 2023-12-21

**Authors:** Soonhyung Kwon, Ellen Benoit, Liliane Windsor

**Affiliations:** Rutgers, the State University of New Jersey, Savoy, Illinois, United States; Center for Drug Use and HIV/HCV Research, NYU School of Global Public Health, New York City, New York, United States; The University of Illinois at Urbana-Champaign, Champaign, Illinois, United States

## Abstract

**Background:**

Social support and self-efficacy play a significant role in improving positive psychological well-being in marginalized older adults. However, to date, there are few studies on identifying the relationships during the COVID-19 pandemic. We examined the effect of social support and self-efficacy on hopefulness in marginalized low-income older adults during the COVID-19 pandemic.

**Methods:**

This study used baseline data from a clinical trial designed to increase COVID-19 testing in Essex County, NJ. The dataset involved participants aged 50 and over. We conducted: 1) cross-sectional descriptive/frequency statistics to understand the sociodemographic characteristics, 2) multivariate linear regression to investigate the direct relationships between social support subscales or self-efficacy and hopefulness, and 3) mediation analyses to examine the mediating role of self-efficacy in the relationship between social support and hopefulness.

**Results:**

After adjusting for covariate variables, social support subscales (emotional/informational social support: b = 0.20, p < 0.05; tangible social support: b = 0.13, p < 0.05; affectionate social support: b = 0.17, p < 0.05; positive social interaction social support: b = 0.15, p < 0.05) and self-efficacy (b = 0.55, p < 0.001) were significantly associated with hopefulness. The indirect effect of social support via self-efficacy was positive and statistically significant (Effect = 0.14, Bootse = 0.04, BootLLCI - BootULCI = 0.06 - 0.23)

**Conclusion:**

Self-efficacy mediated the relationship between social support and hopefulness in marginalized older adults aged 50 and over. Further research needs to identify the various facets of positive psychological well-being using longitudinal data and larger sample size.

